# Parasites and Parasitic Diseases in Small Animals

**DOI:** 10.3390/ani15081183

**Published:** 2025-04-21

**Authors:** Angela M. García-Sánchez, Rocío Callejón

**Affiliations:** Department of Microbiology and Parasitology, Faculty of Pharmacy, University of Seville, Profesor García González 2, 41012 Sevilla, Spain; callejon@us.es

## 1. Introduction

Parasites and parasitic diseases in small animals pose a significant challenge to public health, animal health, and environmental sustainability, and this Special Issue is particularly relevant within the context of the One Health approach, which recognizes their interconnectedness [1].

Humans coexist with animals that are part of our ecosystem, although they are often not visible due to their small size. In addition to animals naturally present in the environment, a large number of small mammals are commonly kept as pets—including dogs, cats, rodents, hedgehogs, and rabbits, not to mention other exotic animals like birds, reptiles, amphibians, and fish. These close relationships explain why some parasitic diseases are also zoonotic, infecting humans and even causing severe illnesses. Zoonoses can also lead to significant economic losses due to decreased productivity and increased healthcare costs. Domestic and wild animals can act as reservoirs and vectors for zoonotic parasites, facilitating disease transmission between species and increasing the risk of outbreaks that compromise both human well-being and the ecological balance [1,2].

Flea infestations and many other parasitic infections are examples that illustrate how parasites can affect multiple hosts in various environments [3,4,5]. These diseases not only cause severe health problems in humans and animals but also lead to significant economic losses due to treatment and control costs, as well as decreased productivity in sectors related to livestock and wildlife management [6]. Furthermore, factors such as climate change, urbanization, and increased human–animal contact have exacerbated the spread of parasites to new geographical regions [6,7].

Although there have been advances in this field in recent years, many aspects remain unknown. The success of the One Health concept now requires breaking down the interdisciplinary barriers that still separate human and veterinary medicine from ecological, evolutionary, and environmental sciences. The surveillance and control of these diseases is essential to mitigate their impact and, without effective strategies that limit transmission from reservoir animals to humans, the optimal control of these infections is not possible. The implementation of measures based on the One Health approach encourages interdisciplinary collaboration among physicians, veterinarians, ecologists, and other specialists to develop integrated solutions that address both the underlying causes and consequences of zoonotic parasitoses [8,9].

In this regard, it is crucial to strengthen epidemiological monitoring initiatives, improve diagnostic tools, and promote educational programs targeted at the general public and health professionals. These actions will not only protect human and animal health but also contribute to maintaining healthy and sustainable ecosystems in an increasingly interconnected world [8,10]. For these reasons, studying and controlling parasitic diseases in small animals is crucial to improving global public health.

## 2. The Structure of This Special Issue

This Special Issue includes eleven papers addressing the challenge of parasitic diseases in small animals, exploring their impact on both wildlife and domestic species. It includes studies on the diversity and host specificity of avian haemosporidians in an Afrotropical conservation region, suggesting that biodiverse areas may harbor a greater variety of parasites, which is crucial for understanding host–parasite dynamics and potential disease emergence in avian populations. On the other hand, the application of geometric morphometrics is introduced to differentiate three populations of synanthropic fleas in Andalusia (Spain), highlighting the importance of this technique for studying and managing arthropod communities.

Further research investigates the detection of β-tubulin polymorphisms in *Trichuris trichiura*, offering promising tools for improving the diagnosis and treatment of trichuriasis. In addition, the prevalence and risk factors of gastrointestinal parasites in domestic dogs in Serbia are explored, providing recommendations to improve parasite control in canines. The Special Issue also covers the occurrence of *Platynosomum illiciens* infection in cats with elevated liver enzymes, shedding light on the epidemiology of this parasitic infection and its effects on feline health.

Additionally, one study examines the status of *Trypanosoma grosi* and *Babesia microti* in small mammals in the Republic of Korea, emphasizing the need to understand the distribution and genetic diversity of these parasites to better manage zoonotic diseases; another reports the detection of *Theileria sinensis*-like and *Anaplasma capra* in ticks, contributing to the knowledge of the distribution and genetic diversity of these pathogens.

The prevalence of *Sarcocystis* spp. macrocysts in wildfowl in the Eastern Baltic region is analyzed over a specific time frame, with a focus on the economic impact of *Sarcocystis rileyi* infections on game birds. Data on new intermediate and accidental hosts of *Angiostrongylus cantonensis* in the Canary Islands (Spain) highlight the parasite’s spread into new areas, underscoring the importance of monitoring its distribution. In addition, the prevalence of microsporidia in the North African hedgehog (*Atelerix algirus*) in the Canary Islands is reported, and its implications for wildlife health and conservation are discussed. Finally, the detection of *Sarcocystis pilosa* in a red fox (*Vulpes vulpes*) reintroduced in South Korea provides insights into the presence of intermediate and definitive hosts in the region, emphasizing the need for monitoring reintroduced wildlife for parasitic infections.

These studies collectively illustrate the complex relationships between parasitic infections, animal health, and ecosystem dynamics.

## 3. Conclusions

This Special Issue highlights a variety of innovative contributions that provide a better understanding of the epidemiology, host specificity, and diversity of parasites and parasitic diseases in small animals. Monitoring and controlling these diseases not only protects animal health but also safeguards public health by reducing the risk of zoonotic infections, due to their close contact with humans, without forgetting the potential outbreaks that may arise in the near future.
